# Expression Profile of Cationic Amino Acid Transporters in Rats with Endotoxin-Induced Uveitis

**DOI:** 10.1155/2016/6586857

**Published:** 2016-06-16

**Authors:** Yung-Ray Hsu, Shu-Wen Chang, Chang-Hao Yang, Yi-An Lee, Tzu-Yun Kao

**Affiliations:** ^1^Department of Ophthalmology, National Taiwan University Hospital, No. 7, Chung-Shan South Road, Taipei 10002, Taiwan; ^2^Department of Ophthalmology, Far Eastern Memorial Hospital, No. 21, Sec. 2, Nanya South Road, Banqiao District, New Taipei City 22056, Taiwan; ^3^Department of Ophthalmology, National Taiwan University Hospital, Yun-Lin Branch, No. 579, Sec. 2, Yunlin Road, Douliou City, Yunlin County 64041, Taiwan

## Abstract

*Purpose*. The transcellular arginine transportation via cationic amino acid transporter (CAT) is the rate-limiting step in nitric oxide (NO) synthesis, which is crucial in intraocular inflammation. In this study, CAT isoforms and inducible nitric oxide synthase (iNOS) expression was investigated in endotoxin-induced uveitis (EIU).* Methods.* EIU was induced in Lewis rats by lipopolysaccharide (LPS) injection. In the treatment group, the rats were injected intraperitoneally with the proteasome inhibitor bortezomib before EIU induction. After 24 hours, leukocyte quantification, NO measurement of the aqueous humor, and histopathological examination were evaluated. The expression of CAT isoforms and iNOS was determined by reverse transcription-polymerase chain reaction, western blotting, and immunofluorescence staining. Nuclear factor-kappa B (NF-*κ*B) binding activity was evaluated by electrophoretic mobility shift assay. The mouse macrophage cell line RAW 264.7 was used to validate the* in vivo* findings.* Results*. LPS significantly stimulated iNOS, CAT-2A, and CAT-2B mRNA and protein expression but did not affect CAT-1 in EIU rats and RAW 264.7 cells. Bortezomib attenuated inflammation and inhibited iNOS, CAT-2A, and CAT-2B expression through NF-*κ*B inhibition.* Conclusions.* CAT-2 and iNOS, but not CAT-1, are specifically involved in EIU. NF-*κ*B is essential in the induction of CAT-2 and iNOS in EIU.

## 1. Introduction

Uveitis is broadly defined as intraocular inflammation. Owing to the complexity of diseases, poorly understood pathogenesis, and multiple disease recurrences, along with prolonged or repeated steroid therapy, uveitis brings about many ocular comorbidities such as cataract, glaucomatous optic neuropathy, or cystoid macular edema [1, 2]. Therefore, uveitis accounts for 10–25% of legal blindness worldwide [3, 4].

Several animal models have been developed to study human uveitis immunopathogenesis. Among them, endotoxin-induced uveitis (EIU) is an animal model of acute anterior segment intraocular inflammation that is induced by the injection of the lipopolysaccharide (LPS) of a Gram-negative bacterial cell wall [5]. Following disease induction, breakdown of the blood-aqueous barrier with cellular infiltration of the iris and ciliary body (ICB) tissue ensues [6, 7]. Immunologically, nuclear factor-kappa B (NF-*κ*B) activation, with the upregulation of numerous downstream proinflammatory cytokines and chemokines, is regarded as being crucial in the disease manifestation of EIU [8, 9]. Because the degradation of ubiquitinated inhibitor of kappa B (I*κ*B) by proteasome is essential in NF-*κ*B activation in the canonical pathway, bortezomib (Velcade), a 26S proteasome inhibitor, inhibits NF-*κ*B expression through the upregulation of I*κ*B [10, 11]. In our previous work, bortezomib reliably reduced the clinical inflammation observed in EIU [12].

Nitric oxide (NO) is an intracellular secondary messenger that serves as an important mediator of homeostasis and normal physiology in the eye and other organs [13]. However, NO overproduction can contribute to intraocular inflammation and ocular tissue destruction [14]. The unique participation of NO in uveitis deserves special attention. First, NO contributes to the breakdown of the blood-aqueous barrier and activates the upregulation of many downstream proinflammatory cytokines, such as TNF-*α* and IL-1. It is thus a potent immunological driver [15]. Second, NO not only induces vasorelaxation but also promotes leukocyte-endothelial cell interactions. These actions all lead to activated neutrophils and monocyte/macrophage aggregation in inflamed tissues [16, 17]. Third, NO and many downstream oxidation products are highly cytotoxic to retinal cells and other ocular tissues. In human sympathetic ophthalmia and some animal models of uveitis, photoreceptor mitochondrial oxidative stress with subsequent apoptosis occurs early in the course of the disease, even in the absence of leukocytic infiltration of the retina [18, 19]. Therefore, NO activation is regarded as a marker for poor disease prognosis in uveitis [20]. Fourth, a close crosstalk and negative feedback loop exists between NF-*κ*B, an important nuclear factor in uveitis, and NO. While NF-*κ*B promotes NO synthase (NOS) upregulation and subsequent NO production, NO in turn inhibits NF-*κ*B expression [21–24].

NO is produced from the essential amino acid L-arginine through the catalytic action of NOS [25, 26]. Transmembrane L-arginine transportation, which is the very first and crucial step of NO production, is mediated by cationic amino acid transporters (CATs) [27, 28]. Among the four categories of CATs, CAT-1, CAT-2A, and CAT-2B are of special significance [29, 30]. CAT-1 is expressed almost ubiquitously except for the liver and is associated with cellular proliferation and cytokine regulation [31, 32]. CAT-2A is most abundant in the liver and various muscular tissues. While the distribution of CAT-2B is similar to that of CAT-2A, CAT-2B expression is associated with inflammatory response [33]. Two NF-*κ*B-binding sites have been identified in the CAT-2 gene promoter region [33, 34]. CAT-2B rises significantly after cytokine or LPS treatment and is often coinduced with induced NOS (iNOS) and CAT-1. Hence, excessive expression of CAT-2, rather than CAT-1, occurs in most systemic inflammatory or infectious processes [35, 36]. However, except for a few studies of ocular surface inflammatory models, research on CATs in intraocular inflammatory diseases is limited [37, 38].

Previous literature has well addressed the involvement of NO in EIU and the effect of iNOS inhibitors in disease amelioration [14, 17, 39, 40]. However, the participation of the key enzyme CAT has not been well studied in EIU. In addition, because there is crosstalk between NF-*κ*B and NO, we hypothesize that the upstream blockade of NF-*κ*B alters the expression pattern of NO and related enzymes and decreases inflammation and tissue destruction. The purpose of this study was hence twofold. First, detailed profiles of NO, CAT-1, CAT-2A, CAT-2B, and iNOS in the ICBs in a rat EIU model were investigated. Second, the effect of the proteasome inhibitor bortezomib on these molecules was analyzed to study the relationship between NF-*κ*B and NO-related enzymology. In addition, as macrophages and neutrophils were regarded as the primary source of NO production in EIU, RAW 264.7, a murine macrophage cell line, was used as an* in vitro* model to confirm the* in vivo* results.

## 2. Materials and Methods

### 2.1. Animal Preparation and Grouping

Lewis rats (8-week-old males; 200–250 g) were used for the experiments. This animal study was approved by the Institutional Animal Care and Use Committee of National Taiwan University Hospital in Taipei, Taiwan. The care and handling of the animals were undertaken in accordance with the ARVO Statement for the Use of Animals in Ophthalmic and Vision Research.

The rats were randomly allocated to four experimental groups, with 20 rats each, as follows. In group 1, the control rats were untreated. In group 2, 300 *μ*g of LPS from* Salmonella typhimurium* (Sigma-Aldrich, St. Louis, MO), in 0.1 mL of sterile saline, was injected into the footpad of the rats to induce EIU. In group 3, the rats were treated with intraperitoneal injections of low-dose bortezomib (0.05 mg/kg; Millennium Pharmaceuticals, Cambridge, MA) 30 min before LPS administration. In group 4, LPS was administered to each rat following high-dose bortezomib (0.2 mg/kg) pretreatment. Bortezomib was diluted with 0.9% sodium chloride saline.

The rats were sacrificed 24 h after disease induction, and their eyes were enucleated for further study. Histological studies and immunofluorescence staining were used to evaluate the inflammatory conditions and CAT and iNOS expression. We also collected the aqueous humor (AqH) to measure the NO concentrations by enzyme-linked immunosorbent assay (ELISA). Reverse transcription-polymerase chain reaction (RT-PCR) and western blotting were performed to evaluate the mRNA and proteins, respectively.

### 2.2. Quantification of Infiltrating Cells in the AqH and Histological Evaluation of the Anterior Segment

Anterior chamber paracentesis was performed with 30-gauge needles to obtain AqH (2 *μ*L). After staining with 0.4% trypan blue, the samples were observed under phase-contrast microscopy to measure the leukocyte numbers.

After enucleation, the eyeballs were immersed in 4% paraformaldehyde in 0.2 M phosphate buffer for 24 h and then dehydrated with alcohol and embedded in paraffin. Sagittal sections (5 *μ*m) near the optic nerve head were obtained, and the specimens were stained with hematoxylin and eosin to evaluate the cellular ICB infiltration.

### 2.3. Immunofluorescence Staining

The sections used for immunofluorescence staining were obtained from the same paraffin blocks and were deparaffinized with xylene solution. After rehydration with a graded series of ethanol in phosphate-buffered saline, 0.3% hydrogen peroxidase was added to block the intrinsic peroxidase activity, and after rinsing with 5% normal rat serum, the specimens were incubated overnight at 4°C with monoclonal antibodies against CAT-1, CAT-2, or iNOS (Santa Cruz Biotechnology, Santa Cruz, CA). FITC-labeled goat anti-rabbit IgG (1 : 200 dilution; Jackson ImmunoResearch, West Grove, PA) was used as the secondary antibody. The specimens were examined using fluorescence microscopy, and the same exposure was used to photograph all of the samples. All of the slices for the immunofluorescence staining of CAT-1, CAT-2, and iNOS were also counterstained with 4′,6-diamidino-2-phenylindole (DAPI).

### 2.4. Cell Culture and Treatment

Mouse macrophage RAW 264.7 cells were obtained from the American Type Culture Collection (Manassas, VA; Rockville, MD). All of the cells were grown in RPMI-1640 medium at 37°C in a humidified incubator containing 5% CO_2_.

The RAW 264.7 cells were divided into four groups. Group 1 was the control group. The cells in group 2 were stimulated with 10 ng/mL LPS from* Salmonella typhimurium* (Sigma-Aldrich) for 30 min. The cells in group 3 were pretreated with low-dose bortezomib (10 nM) for 2 h and then stimulated with 10 ng/mL LPS for 30 min. The cells in group 4 were pretreated with high-dose bortezomib (25 nM) for 2 h and then stimulated with 10 ng/mL LPS for 30 min.

### 2.5. Semiquantitative RT-PCR

iNOS, CAT-1, CAT-2, CAT-2A, and CAT-2B mRNA expression from the ICB tissues and RAW 264.7 cells were measured using semiquantitative RT-PCR. Total RNA was extracted with TRIzol reagent (Invitrogen-Life Technologies, Inc., Gaithersburg, MD). Total RNA (1 *μ*g) from each sample was annealed with 300 ng of oligo (dT) (Promega, Madison, WI) for 5 min at 65°C. The housekeeping gene *β*-actin was used as an internal standard. We used 80 U of Moloney murine leukemia virus reverse transcriptase (MMLV-RT; Invitrogen-Gibco, Grand Island, NY) per 50 *μ*g sample for 1 h at 37°C, and the reaction was stopped by increasing the temperature to 90°C for 5 min. The resultant cDNA from each sample was subjected to PCR with specific primers (see Table 1). The reaction mixture (50 *μ*L) contained 5 mL of cDNA, 1 *μ*L of sense and antisense primers, 200 *μ*M of each deoxynucleotide, 5 *μ*L of 10x* Taq* polymerase buffer, and 1.25 U of* Taq* polymerase (Promega). A thermocycler (MJ Research, Waltham, MA) with 1 min denaturation at 94°C and 3 min extension at 72°C was used for amplification. The annealing temperature was between 62°C and 42°C, and the temperature was decreased in 1°C increments, followed by 21 cycles at 55°C. Finally, the temperature was elevated to 72°C for 10 min and then reduced to 4°C. Samples (10 mL) of each PCR product were obtained for gel electrophoresis on 2% agarose gels containing ethidium bromide (Sigma-Aldrich). A DNA molecular length marker with ultraviolet light was used for analyzing the results. Image analysis software (Photoshop, version 7.0; Adobe Systems, San Jose, CA) was used to determine the intensity. All of the experiments were repeated three times, and they all yielded similar results.

### 2.6. Western Blotting

The ICB tissues and RAW 264.7 cells were also tested by western blotting to study the iNOS, I*κ*B, CAT-1, CAT-2, and *β*-actin protein expression. The ICB tissues were treated with radioimmunoprecipitation assay buffer [0.5 M Tris-HCl (pH 7.4), 1.5 M NaCl, 2.5% deoxycholic acid, 10% NP-40, 10 mM EDTA, and protease inhibitors (Complete Mini; Roche Diagnostics Corp., Indianapolis, IN)] to extract the total protein. A mixture of extract and Laemmli sample buffer (1 : 1 ratio) was boiled for 5 min. We also extracted the cellular proteins from the RAW 264.7 cells with Laemmli sample buffer for further study. Each sample (100 *μ*g) was separated on 10% SDS-polyacrylamide gels and was then transferred to polyvinylidene difluoride membranes (Immobilon-P; Millipore Corp., Billerica, MA), which were incubated with anti-iNOS, anti-I*κ*B, anti-CAT-1, anti-CAT-2, and anti-*β*-actin antibodies. The membranes were visualized by chemiluminescence (GE Healthcare, Buckinghamshire, UK) after incubation with horseradish peroxidase-conjugated secondary antibody, and the density of the blots was quantified with the assistance of image analysis software after scanning the image (Photoshop, version 7.0; Adobe Systems). The optical densities of each band were calculated and standardized based on the *β*-actin band density. All of the experiments were performed independently three times, and they all yielded similar results.

### 2.7. ELISA

The NO concentrations in the AqH and cell culture medium were determined using commercial ELISA kits (R&D Systems, Minneapolis, MN) according to the manufacturer's instructions. The samples were diluted to 50 *μ*L for testing, and the optical density was determined at *A*_450_ (absorbance at 450 nm) using a microplate reader (Bio-Rad, Hercules, CA).

### 2.8. Nuclear Protein Extraction and NF-*κ*B Electrophoretic Mobility Shift Assay (EMSA)

The ICB tissues and RAW 264.7 cells were trypsinized, resuspended, and homogenized in buffer A [10 mM HEPES (pH 7.9), 1.5 mM KCl, 10 mM MgCl_2_, 1.0 mM dithiothreitol (DTT), and 1.0 mM phenylmethylsulfonyl fluoride (PMSF)]. The tissue was then homogenized (Dounce; Bellco Glass Co., Vineland, NJ), followed by centrifugation at 5000 ×g at 4°C for 10 min. After suspension in 200 mL of buffer B [20 mM HEPES (pH 7.9), 25% glycerol, 1.5 mM MgCl_2_, 420 mM NaCl, 0.5 mM DTT, 0.2 mM EDTA, 0.5 mM PMSF, and 4 *μ*M leupeptin], the sample was incubated on ice for 30 min and centrifuged at 12000 ×g at 4°C for 30 min. The supernatant containing the nuclear proteins was collected and stored at −70°C until use.

A bicinchoninic acid assay kit, with bovine serum albumin as the standard (Pierce Biotechnology, Rockford, IL), was used to determine the protein concentration. EMSA was performed with an NF-*κ*B DNA-binding protein-detection system (Pierce Biotechnology) according to the manufacturer's instructions. We incubated a 10 *μ*g nuclear protein aliquot with a biotin-labeled NF-*κ*B consensus oligonucleotide probe (5′-AGTTGAGGGGACTTTCCCAGGC-3′) for 30 min in binding buffer and then determined the specificity of the DNA/protein binding by adding 100-fold molar excess of unlabeled NF-*κ*B oligonucleotide for competitive binding 10 min before adding the biotin-labeled probe.

### 2.9. Statistical Analysis

All of the data are expressed as means ± SDs. We analyzed our data with the nonparametric Mann-Whitney *U* test. The significance level was set at 0.05. A commercial software package (SigmaStat for Windows; SPSS Science, Chicago, IL) was used for the data analysis.

## 3. Results

### 3.1. Histopathologic Study of Inflammation in EIU

**Figure 1:**
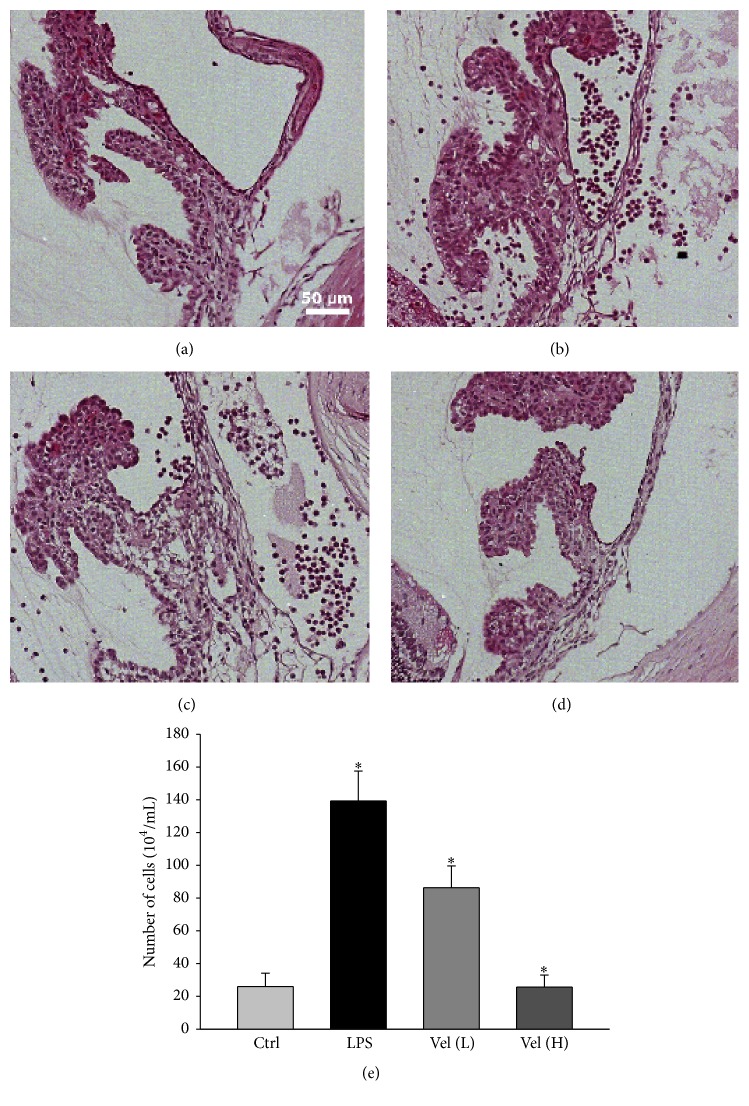
Histological evaluation of cellular infiltration into the iris and ciliary bodies. Vel (L) = low-dose bortezomib, Vel (H) = high-dose bortezomib; inflammatory cell infiltration can be observed in the ciliary bodies of (b) LPS only and (c) low-dose bortezomib-treated rat eyes but not in (a) the control and (d) high-dose bortezomib-treated groups. Scale bar = 50 *μ*m. (e) Quantification of the infiltrating cells in the AqH. EIU rats pretreated with bortezomib showed significantly reduced cell numbers in the AqH in a dose-dependent manner. The AqH was pooled from one eye of five rats in each group. The data are expressed as the means ± SDs of three independent experiments (bar graph). ^*∗*^ *p* < 0.05 compared with the control group.

Histological sections of the anterior chamber demonstrated little inflammatory cell infiltration in the control group or the high-dose bortezomib group (Figures 1(a) and 1(d)). In contrast, the ICBs of rats in the LPS group and low-dose bortezomib group were infiltrated with large numbers of inflammatory cells (Figures 1(b) and 1(c)). We also demonstrated statistically significant differences of the cell counts among the different treatment groups (Figure 1(e)). The number of inflammatory cells in the LPS group was significantly higher than that in the control group (*p* < 0.05). Dose-dependent pretreatment of rats with the proteasome inhibitor bortezomib decreased the inflammatory cell numbers in the AqH.

### 3.2. Immunofluorescence Staining for ICB CAT and iNOS

**Figure 2:**
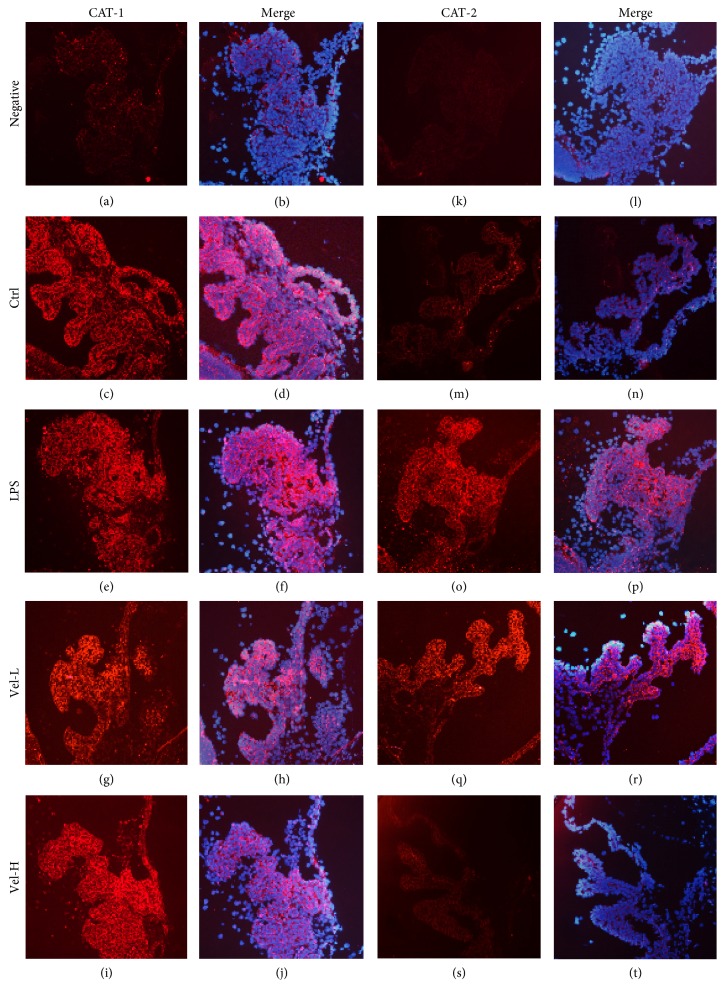
Expression of CAT in the iris and ciliary bodies detected by immune-fluorescence staining. Vel-L = low-dose bortezomib; Vel-H = high-dose bortezomib; CAT-1 and CAT-2 were immunostained as red. In the “Merge” column (b, d, f, h, j and l, n, p, r, t), all of the slices were counterstained with DAPI (4′,6-diamidino-2-phenylindole) that stains blue for better iris-ciliary body tissue visualization. CAT-1 was extensively expressed in the ICBs of all the treatment groups (c, e, g, and i), whereas CAT-2 was expressed only in the LPS- (o) and low-dose bortezomib-treated groups (q). High-dose bortezomib (s) treatment reduced the CAT-2 expression in the ICBs of EIU rats. Scale bar = 50 *μ*m.

**Figure 3:**
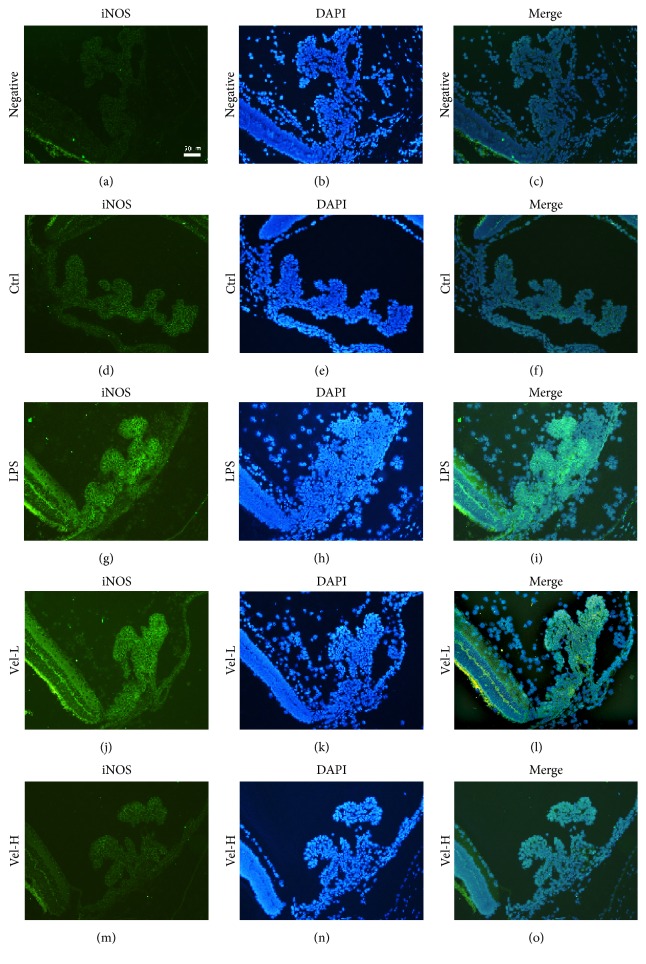
Expression of iNOS in the iris and ciliary bodies detected by immunofluorescence staining. Vel-L = low-dose bortezomib; Vel-H = high-dose bortezomib; iNOS was expressed in green, while the counterstain DAPI (4′,6-diamidino-2-phenylindole) was expressed in blue. iNOS was only expressed in the LPS- (g) and low-dose bortezomib-treated (j) groups. Treatment with high-dose bortezomib (m) reduced the iNOS expression in the ciliary bodies of EIU rats. Scale bar = 50 *μ*m.

The expression of CAT-1 was observed in all treatment groups (Figures 2(c)–2(i)). In contrast, CAT-2 expression was only found in the LPS (Figure 2(o)) and low-dose bortezomib groups (Figure 2(q)). There was no obvious CAT-2 expression in the control (Figure 2(m)) or high-dose bortezomib groups (Figure 2(s)). The ICB iNOS expression was similar to that of CAT-2. There was no obvious expression of iNOS in the control (Figure 3(d)) or high-dose bortezomib groups (Figure 3(m)), but the expression of iNOS was found in the LPS (Figure 3(g)) and low-dose bortezomib groups (Figure 3(j)). The slices counterstained with DAPI confirmed these results (Figures 2(b)–2(j), 2(l)–2(t), and 3(c)–3(o)).

### 3.3. ICB CAT and iNOS mRNA Expression

**Figure 4:**
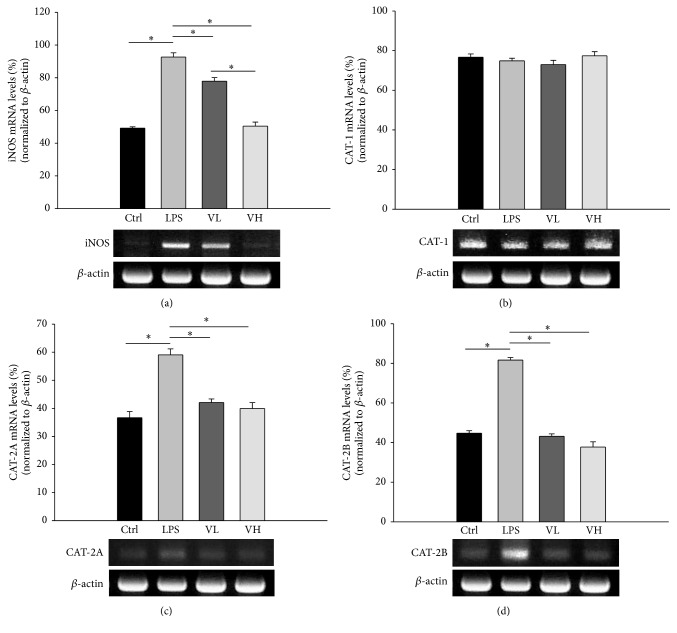
Expression of mRNA of CAT-1, CAT-2A, CAT-2B, and iNOS in ICBs with semiquantitative RT-PCR. The RNA was isolated under control conditions (Ctrl), in rats treated with LPS (LPS), and rats pretreated with low-dose bortezomib (VL) or high-dose bortezomib (VH). LPS induced the expression of iNOS, CAT-2A, and CAT-2B mRNA in the ICBs of EIU rats. Pretreatment with bortezomib decreased their expression (a, c, and d). There were no significant differences in CAT-1 mRNA levels among the four groups (b). The symbol “*∗*” indicates statistical significance.

CAT-1, CAT-2A, CAT-2B, and iNOS mRNA expression in the EIU rat ICBs was evaluated by semiquantitative RT-PCR. The iNOS mRNA level was significantly elevated in the ICB after treatment with LPS and significantly decreased in the rats treated with bortezomib (*p* < 0.05 for these comparisons; Figure 4(a)). There were no significant differences in the CAT-1 mRNA levels among the four groups (Figure 4(b)). The CAT-2A and CAT-2B mRNA levels in the LPS group were significantly higher than those in the control group. In the low- and high-dose bortezomib groups, the CAT-1A and CAT-1B mRNA levels were significantly lower than those in the LPS group (*p* < 0.05 for these comparisons; Figures 4(c) and 4(d)).

### 3.4. ICB CAT and iNOS Protein Levels

**Figure 5:**
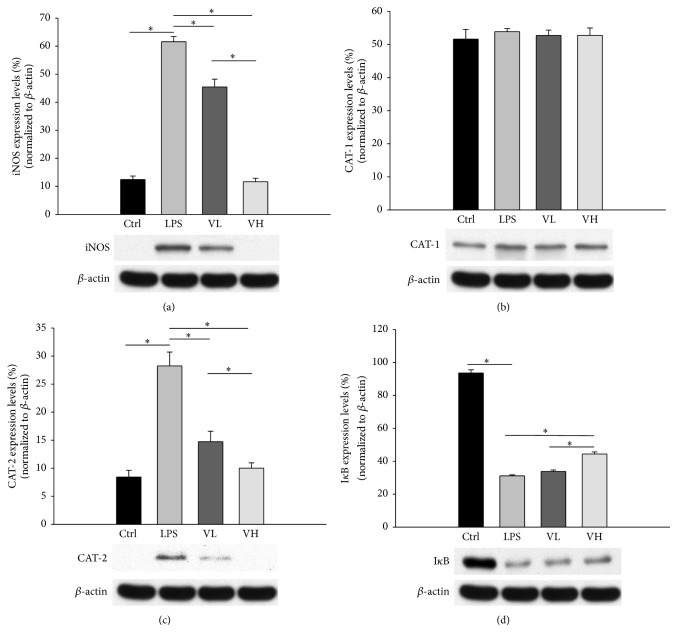
Expression of iNOS, CAT-1, and CAT-2 proteins in the ICBs as assessed by western blotting. The proteins were isolated under control conditions (Ctrl), from rats injected with LPS (LPS), and from rats pretreated with low-dose bortezomib (VL) or high-dose bortezomib (VH). LPS induced the expression of iNOS and CAT-2 proteins in the ICBs of EIU rats. Pretreatment with bortezomib decreased their expression (a and c). There were no significant differences in CAT-1 protein levels among the four groups (b). As compared with the control group, the protein levels of I*κ*B decreased in the LPS group and increased in the bortezomib treatment groups. The symbol “*∗*” indicates statistical significance.

iNOS, CAT-1, and CAT-2 protein expression in the EIU rat ICBs was evaluated by western blotting. The iNOS protein level in the LPS group was significantly higher than that in the control group. In the low- and high-dose bortezomib-treated groups, the iNOS protein levels demonstrated a significant dose-dependent decrease as compared with that of the LPS group (*p* < 0.05 for these comparisons; Figure 5(a)). The CAT-1 protein levels were similar in the four groups (Figure 5(b)). The CAT-2 protein levels in the LPS group were significantly higher than those in the control group and decreased in a dose-dependent manner after bortezomib treatment (*p* < 0.05 for these comparisons; Figure 5(c)). The I*κ*B protein levels significantly decreased in the LPS group as compared with those in the control group and significantly increased in the bortezomib treatment group (*p* < 0.05 for these comparisons; Figure 5(d)).

### 3.5. CAT and iNOS mRNA Expression in RAW 264.7 Cells

**Figure 6:**
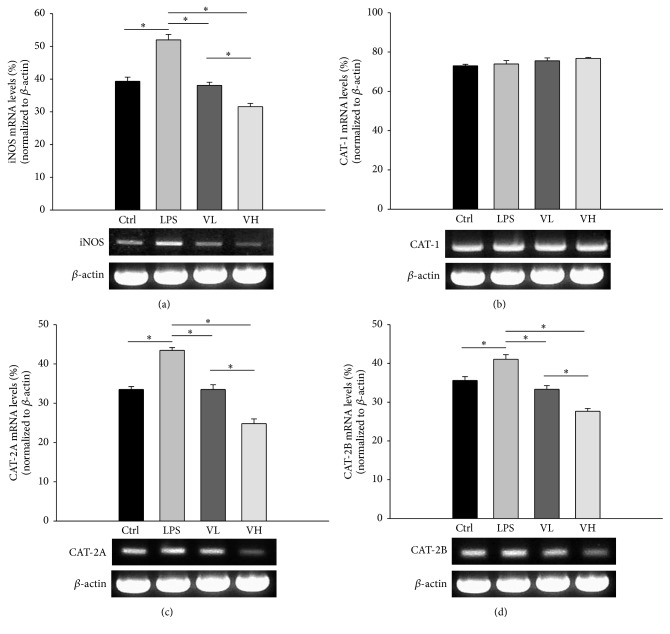
CAT and iNOS mRNA expression in RAW 264.7 cells. The RNA was isolated under control conditions (Ctrl), from rats treated with LPS alone (LPS), and from rats pretreated with 10 nM bortezomib (VL) or 25 nM bortezomib (VH). The symbol “*∗*” indicates statistical significance.

iNOS, CAT-2A, and CAT-2B mRNA expression in RAW 264.7 cells was significantly higher in the LPS group than in the control group, and their expression decreased in the low- and high-dose bortezomib groups (*p* < 0.05 for these comparisons; Figures 6(a), 6(c), and 6(d)). The levels of CAT-1 mRNA expression in RAW 264.7 cells were similar in the four groups (Figure 6(b)).

### 3.6. CAT, iNOS, and I*κ*B Protein Levels in RAW 264.7 Cells

**Figure 7:**
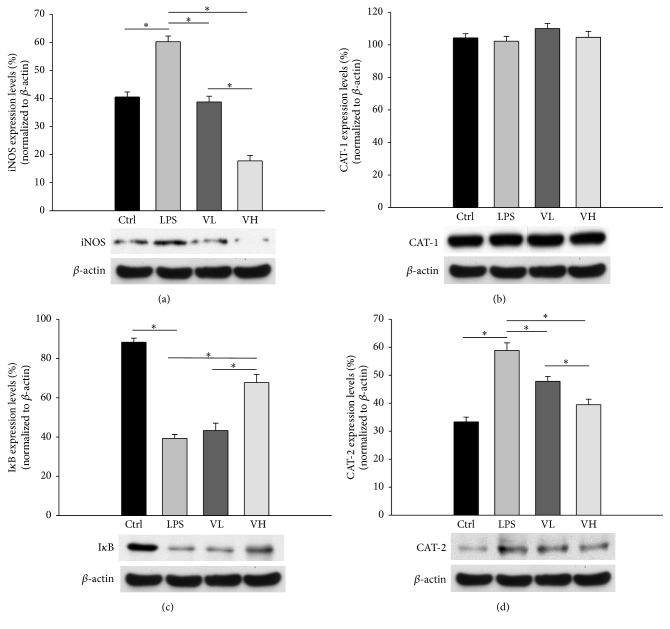
CAT, iNOS, and I*κ*B protein levels in RAW 264.7 cells. The cells were grouped into control conditions (Ctrl), treatment with LPS (LPS), and pretreatment with 10 nM bortezomib (VL) or 25 nM bortezomib (VH). The protein levels of iNOS, I*κ*B, CAT-1, and CAT-2 were analyzed by western blotting. The symbol “*∗*” indicates statistical significance.

The protein levels of iNOS and CAT-2 in RAW 264.7 cells significantly increased in the LPS group as compared with those in the control group and decreased in a dose-dependent manner in the bortezomib treatment groups (Figures 7(a) and 7(d)). There were no significant differences in the CAT-1 protein levels in RAW 264.7 cells among the four groups (Figure 7(b)). The I*κ*B protein levels in RAW 264.7 cells significantly decreased in the LPS group as compared with those in the control group and increased in a dose-dependent manner in the bortezomib treatment groups (Figure 7(c)).

### 3.7. NO Concentration in the AqH of EIU Rats and in the Medium of RAW 254.7 Cells

**Figure 8:**
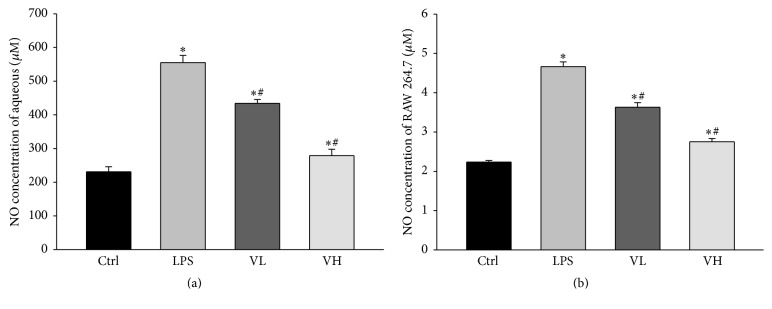
The concentrations of NO in the aqueous humor as measured by ELISA. The concentrations of NO in the aqueous humor of EIU rats (a) and in RAW 264.7 cells treated with LPS (b). The aqueous humor was isolated under control conditions (Ctrl), pretreatment with LPS (LPS), and pretreatment with low-dose bortezomib (VL) or high-dose bortezomib (VH). LPS injection increased the NO production in the aqueous humor of EIU rats, and treatment with bortezomib significantly decreased the NO concentration in the aqueous humor in a dose-dependent manner. The symbols “*∗*” and “#” indicate statistical significance.

Data from ELISA revealed that the NO concentration in the AqH was significantly higher in the LPS group than in the control group. Treatment with bortezomib significantly decreased the NO concentration in the AqH in a dose-dependent manner (Figure 8(a)). Treatment of RAW 264.7 cells with LPS significantly increased the NO concentration in the culture medium as compared with that in the control group. The NO concentration significantly decreased in the low- and high-dose bortezomib groups in a dose-dependent manner (Figure 8(b)).

### 3.8. DNA-Binding Activity of NF-*κ*B in the ICB of EIU Rats and in RAW 264.7 Cells

**Figure 9:**
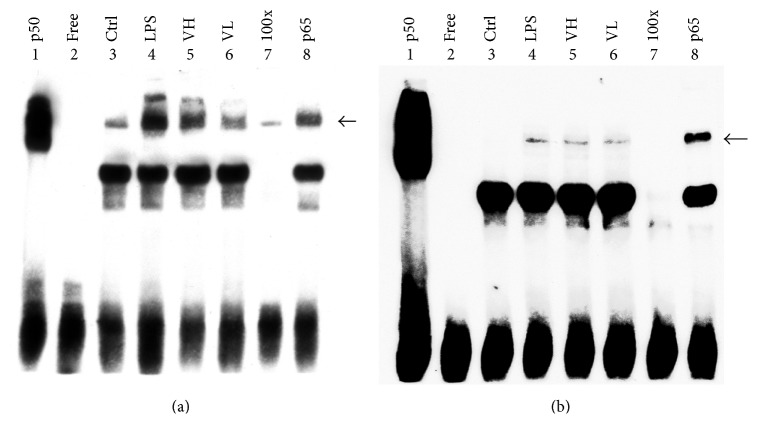
Electrophoretic mobility shift assay to study DNA-binding activity. Electrophoretic mobility shift assay was performed to study the DNA-binding activity of NF-*κ*B in the ICB of EIU rats (a) and in RAW 264.7 cells treated with LPS (b). The results showed that the DNA-binding activity increased in the LPS group, and treatment with bortezomib effectively lowered the NF-*κ*B activity. Lane 1: p50 subunit of NF-*κ*B. Lane 2: free probe. Lane 3: control group. Lane 4: LPS-treated group. Lane 5: LPS with high-dose bortezomib. Lane 6: LPS with low-dose bortezomib. Lane 7: competition with 100x unlabeled NF-*κ*B probe. Lane 8: anti-p65 antibody supershift band.

The DNA-binding activity of NF-*κ*B in the ICBs of EIU rats and in RAW 264.7 cells treated with LPS was evaluated by EMSA, and the results are shown in Figure 7. The DNA-binding activity of NF-*κ*B was higher in the LPS group than in the control group. In contrast, the NF-*κ*B activity decreased in the bortezomib treatment groups in a dose-dependent manner (Figures 9(a) and 9(b)).

## 4. Discussion

In general, the results from the present study provided some important information on EIU pathogenesis. Both CAT-1 and CAT-2 expression in ICB tissue were demonstrated histopathologically in a rat EIU model. Moreover, the finding of simultaneous iNOS and CAT-2 but not CAT-1 upregulation in EIU eyes revealed the different biological nature between CAT-1 and CAT-2. iNOS and CAT-2 expression is responsive to inflammation, but CAT-1 expression is constitutive. NF-*κ*B is an essential transcription factor for CAT-2 and iNOS induction, and inhibition of NF-*κ*B substantially decreased the enzymes' expression and NO release. In addition to the* in vivo* model, the RAW 264.7 cell line study revealed similar results.

The roles of CAT in numerous organs have been well investigated [41, 42]. For instance, CAT-2 is highly upregulated in experimental asthma and is critically important in interstitial lung fibrosis [43]. iNOS and CAT-2 expression is also strongly associated with cardiac myocyte and renal cell inflammation [27, 41, 44]. Regarding ocular tissues, CAT-1 and CAT-2 have been localized in healthy lacrimal glands, conjunctiva, cornea, and nasolacrimal ducts [45]. Physiologically, CAT-1 has been found to be responsible for molecular or nutrient transport in ocular surfaces [37], across lens epithelial cells [46], and the inner blood-retinal barrier [31, 47]. Owing to the transport ability of CATs, it has also been suggested that amino acid prodrugs utilizing CAT transport may improve the solubility of poorly aqueous soluble compounds [38, 48]. However, there is scarce pathological evidence of CAT involvement in ocular inflammatory conditions. Jager et al. demonstrated that CAT-1 activity does not change in inflammatory ocular surface conditions such as keratoconus, Fuchs' dystrophy, and herpetic keratitis, and CAT-2 increases significantly in these tissues and in response to the stimulation by the supernatant of* Pseudomonas aeruginosa* [45]. To the best of our knowledge, the present study is the first one to demonstrate that CAT-2 and iNOS are simultaneously upregulated in uveitis. The fact that only CAT-2, but not CAT-1, markedly increases upon stimulation in EIU is consistent with the current notion that CAT-2 plays an active role in NO production in inflammation. CAT-2 may therefore serve as a potential therapeutic target in future.

CAT-2 has two alternatively spliced transcripts: CAT-2A and CAT-2B. In cardiac myocytes and vascular smooth muscle cells, both CAT-2A and CAT-2B are induced by inflammatory stimuli [36, 49]. However, in rat liver and glomeruli, only CAT-2B, but not CAT-2A, expression is affected by LPS stimulation, which indicates a strong association with inflammation [44, 50]. Using a hybrid depletion study in* Xenopus* oocytes, Kakuda et al. found that bacterial toxins upregulated CAT-2B expression in activated macrophages [51]. In our study, we found that both CAT-2A and CAT-2B were upregulated following LPS stimulation in ICB tissue and RAW 264.7 cells. This discrepancy might be due to the different cell types and disease models studied.

Numerous studies have attempted to investigate the role of NO and the associated enzymology expression following NF-*κ*B inhibition [52–55]. For example, Yang et al. showed that inhibition of NF-*κ*B resulted in cytoprotective effects against sepsis in rat livers associated with CAT-2 mRNA downregulation [50]. Chtourou and associates demonstrated the antiapoptotic and anti-inflammatory effects of naringin on cisplatin-induced renal injury in rats through inhibition of NF-*κ*B and downstream iNOS pathways [54]. Meanwhile, some molecules that exhibit inhibitory effects on NF-*κ*B have also been used therapeutically in experimental uveitis models. Among them, iNOS activation and NO production can be diminished reliably through NF-*κ*B blockade [12, 56, 57]. However, CAT-1 and CAT-2, which are the crucial enzymes responsible for NO production, have not received much attention. The present study enhances our understanding of NF-*κ*B inhibition in the treatment of intraocular inflammation and possibly other systemic inflammatory disease entities, by both attenuating inflammation and reducing oxidative stress. Detailed enzyme profiling delineates the different participation of CAT-1, iNOS, CAT-2A, and CAT-2B in the disease model.

In conclusion, we demonstrated the existence of CAT-1 and CAT-2 expression in intraocular tissue. CAT-1 was constitutively expressed, and CAT-2 expression was strongly associated with inflammation. NF-*κ*B actively participated in iNOS and CAT-2 induction, while NF-*κ*B inhibition attenuated NO production through iNOS and CAT-2 inhibition and, in turn, significantly reduced endotoxin-induced ocular inflammation.

## Figures and Tables

**Table 1:** Sequences of primers used in RT-PCR.

Name of primers	Sequences	Product size (bp)
*β*-actin	5′-CTGGAGAAGAGCTATGAGCTG-3′	246
	5′-AATCTCCTTCTGCATCCTGTC-3′	

iNOS	5′-AGA AGC AGA ATG TGA CCA TC-3′	369
	5′-ACT TCC CTG TCT CAG TAG CA-3′	

CAT-1	5′-TAG GAC CAA AAC ACC CGT AA-3′	388
	5′-GGT GAT GAT AAG AGC GGC TA-3′	

CAT-2A	5′-TGT TTG CTT TAT GGC CTA TT-3′	277
	5′-GTT TTG GAA TTG ATT TGA GC-3′	

CAT-2B	5′-TGT TTG CTT TAT GGC CTA TT-3′	365
	5′-GGG TAA GGG GAA CAT AGA AC-3′	

iNOS: inducible nitric oxide synthase; CAT: cationic amino acid transporter.
